# Swt21p Is Required for Nam8p-U1 snRNP Association and Efficient Pre-mRNA Splicing in *Saccharomyces cerevisiae*

**DOI:** 10.3390/ijms26125440

**Published:** 2025-06-06

**Authors:** Ke Lin, Xiuhu Fu, Lulu Wang, Sa Xiao, Shenxin Wang, Yingjie Fan, Xinyu An, Kum-Loong Boon, Penghui Bao

**Affiliations:** Institute of Molecular Enzymology, School of Life Sciences, Suzhou Medical College of Soochow University, Suzhou 215123, China; 20214021005@stu.suda.edu.cn (K.L.); 20224021019@stu.suda.edu.cn (X.F.); luluwang66546@163.com (L.W.); xiaosa666g@163.com (S.X.); 20224221033@stu.suda.edu.cn (S.W.); 20224221010@stu.suda.edu.cn (Y.F.); 20244221039@stu.suda.edu.cn (X.A.); boonkumloong@yahoo.com (K.-L.B.)

**Keywords:** Swt21p, spliceosome assembly, U1 snRNP, Nam8p, pre-mRNA splicing

## Abstract

While the U1 small nuclear ribonucleoprotein (snRNP) plays a crucial role in early spliceosome assembly, the mechanisms by which it coordinates with other splicing factors for efficient assembly remain elusive. This study aimed to examine the role of the Swt21 protein in regulating U1 snRNP in *Saccharomyces cerevisiae*. Swt21p was required for efficient pre-mRNA splicing both in vivo and in vitro. Deletion of *SWT21* altered the splicing patterns of two-intron *SUS1* RNA, causing intron retention and exon skipping. Spliceosome assembly analysis revealed that in the pre-B complex, the levels of U1 protein components, as well as U1 RNA, were decreased following *SWT21* deletion, highlighting the compromised stability of U1 snRNP during this stage. Consistently, in the absence of Swt21p, free isoform of U1 component Nam8p was observed, and its proper nuclear localization was disrupted, demonstrating the functional importance of Swp21p for the stable association of Nam8p with U1 snRNP. Moreover, Swt21p remained primarily in a free state under physiological conditions and did not associate with the pre-B complex. Additionally, TAP analysis revealed that Swt21p-associated proteins are involved in cellular processes beyond splicing. These findings collectively indicate that Swt21p functions as a spliceosome regulator rather than a core component and support a model wherein Swt21p contributes to U1 snRNP stability during early spliceosome assembly.

## 1. Introduction

As is well documented, pre-mRNA splicing is an essential step in eukaryotic gene expression, where introns are excised and exons ligated to generate mature mRNA [1]. This process is catalyzed by the spliceosome, a macromolecular complex consisting of uridine-rich snRNPs (U1, U2, U4, U5, and U6) and other non-snRNP proteins [2,3]. The spliceosome assembles dynamically, ensuring accurate splicing in a stepwise manner. During spliceosome assembly, U1 snRNP initially recognizes the 5′ splice site (5′ss), and U2 snRNP interacts with the branch point site (BPS) to form the A complex. Subsequently, the U4/U6.U5 tri-snRNP is recruited, forming the fully assembled pre-B complex, which establishes the structural foundation necessary for catalysis [4,5,6,7]. With advancements in cryo-EM technology, numerous spliceosome intermediates have been resolved at atomic resolution, dramatically enhancing our understanding of splicing mechanisms [8]. However, ascribed to its dynamic nature and the involvement of regulatory factors, the precise mechanisms that ensure spliceosome assembly efficiency remain largely unknown.

Noteworthily, U1 snRNP plays a critical role in initiating spliceosome assembly, a process mediated by base-pairing between U1 snRNA and the 5′ss, along with protein-RNA interactions [9]. In *Saccharomyces cerevisiae*, U1 snRNP contains the seven-membered Sm ring, three conserved U1-specific subunits (Snp1, Mud1, and Yhc1, homologous to human U1-70K, U1-A, and U1-C, respectively), as well as seven additional subunits (Nam8p, Snu71p, Snu56p, Luc7p, Prp40p, Prp42p, and Prp39p) [10,11,12]. These additional components facilitate U1 snRNP recruitment to the 5′ss, which plays a central role in the recognition of non-consensus splice sites or in the presence of mutations in U1-associated proteins or snRNA [13,14]. In addition to its constitutional components, U1 snRNP function is regulated by auxiliary factors during spliceosome assembly. In humans, these regulatory mechanisms include post-translational modifications, protein–protein interactions, and phase separation [15,16,17]. For instance, U1-70K phosphorylation acts as a switch that modulates U1 snRNP’s interaction with SR proteins and drives early spliceosome assembly [15]. However, in yeast, the specific regulatory mechanisms for U1 snRNP have not yet been fully elucidated and warrant further investigation.

In this context, Swt21p (also referred to as Ynl187w) is a fungus-specific nuclear protein that has been shown to facilitate interactions between U1 snRNP and the 5′ss [18], although its precise molecular function in spliceosome assembly remains unclear. Swt21p was originally implicated in splicing due to its interaction with SmBp, identified in a large-scale two-hybrid screen [19]. Subsequent studies have reported genetic interactions between Swt21p with Prp40p [20] and Nam8p through two separate synthetic lethality screens [21], suggesting a potential functional link between Swt21p and U1 snRNP.

Here, we applied spliceosome affinity purification, glycerol gradient analysis, and tandem affinity purification (TAP) to dissect the role of Swt21p in U1 snRNP regulation during early spliceosome assembly. The results reveal that *SWT21* deletion leads to a decrease in U1 snRNP levels, suggesting a disruption in pre-B complex assembly. Furthermore, Swt21p stabilizes Nam8p binding to U1 snRNP and is integral for the proper nuclear localization of Nam8p. Moreover, the findings conjointly suggest that Swt21p serves as a key regulatory factor in spliceosome assembly rather than as a core component. Overall, these findings highlight the functional importance of Swt21p in regulating U1 snRNP and provide novel insights into fungal-specific splicing mechanisms.

## 2. Results

### 2.1. Swt21p Affects the Efficiency of Pre-mRNA Splicing In Vivo and In Vitro

To investigate the function of Swt21p, a *swt21*Δ strain was generated in *S. cerevisiae* by deleting the *SWT21* gene. Initially, potential phenotypic changes were assessed by comparing the growth of *swt21*Δ and wild-type strains under various temperature conditions. Interestingly, no significant differences in growth were observed (Figure S1A), suggesting that *SWT21* deletion did not affect yeast growth. Although Swt21p has been previously implicated in stabilizing U1 snRNP/5′ss interactions [18], its impact on pre-mRNA splicing remains to be determined. Consequently, the splicing efficiency of diverse pre-mRNAs was examined in the *swt21*Δ strain. While the majority of *S. cerevisiae* genes contain only one intron with conserved splice sites, some genes feature multiple introns or non-consensus splice sites [22].

**Figure 1:**
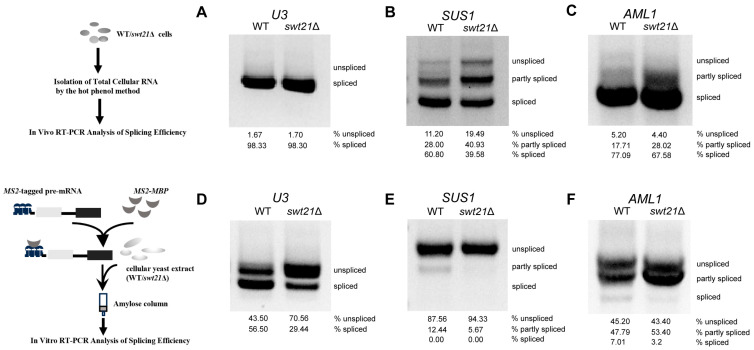
Swt21p affects pre-mRNA splicing in vivo and in vitro. The left panel displays schematic representations of the experimental workflows for in vivo (top) and in vitro (bottom) splicing assays. (**A**–**C**) Swt21p promotes efficient multi-intron splicing in vivo. RNA from wild-type (WT) and *swt21*Δ strains was reverse-transcribed using random primers, and cDNA was amplified by PCR with gene-specific primers for the first and last exons of *U3* (**A**), *SUS1* (**B**), and *AML1* (**C**). PCR products were separated by native agarose gel electrophoresis and detected using Gel Red staining. (**D**–**F**) Swt21p functions as a general splicing factor in vitro. Splicing reactions were performed at 23 °C using the wild-type and *swt21*Δ extracts. RNA was phenol–chloroform-extracted and analyzed by RT-PCR. The unspliced, partly spliced, and fully spliced RT-PCR products are labeled on the right. Percentages of each splicing product were quantified and are displayed below each corresponding lane.

For in vivo splicing efficiency analysis, total RNA from wild-type and *swt21*Δ strains was analyzed by RT-PCR using primers flanking the first and last introns. Agarose gel electrophoresis was employed to distinguish unspliced pre-mRNAs (longer fragments) from mature spliced mRNAs or partially spliced intermediates (shorter fragments). The results showed that *SWT21* deletion specifically impaired the splicing efficiency of multiple-intron genes, such as *SUS1*, *AML1* (Figure 1B,C), and *DYN2* (Figure S1D), as evidenced by alterations in the ratios of unspliced, partially spliced, and fully spliced transcripts. In contrast, no significant differences were observed in the splicing of single-intron genes, such as *U3* (non-canonical BPS), *GLC7* (canonical splice site), and *YFR045W* (non-canonical 5′ss and BPS) in the *swt21*Δ strain (Figure 1A; Figure S1B,C). Taken together, these results signal that the splicing defect observed in *swt21*Δ was more evident for multi-intron genes in vivo.

Based on these results, in vitro splicing reactions were conducted using extracts from wild-type and *swt21*Δ yeast cells. These assays revealed a broader role of Swt21p in splicing, given that its deletion variably impaired the splicing of multiple pre-mRNAs. In *swt21*Δ extracts, mature mRNA levels of *U3* and *AML1* were reduced, and a partially spliced intermediate accumulated in *AML1* (Figure 1D,F). For *SUS1*, which exhibited low splicing efficiency under wild-type conditions, *SWT21* deletion resulted in an almost complete loss of both intermediate and mature RNA species (Figure 1E). The observed differences in splicing efficiency between in vivo and in vitro conditions upon *SWT21* deletion may result from the involvement of redundant factors in vivo.

### 2.2. The Pattern of SUS1 Splicing Is Altered upon swt21Δ

**Figure 2:**
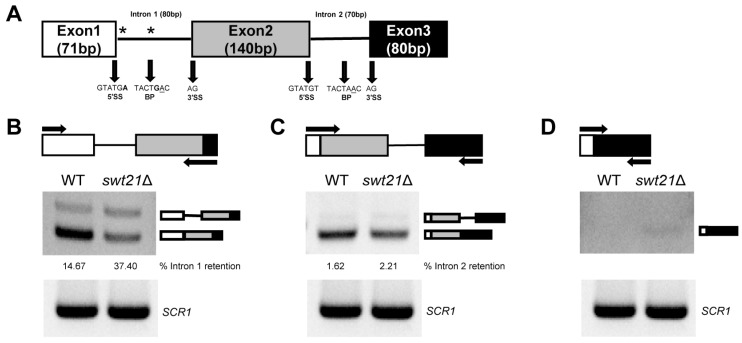
*SWT21* deletion alters the splicing patterns of two-intron *SUS1* RNA. (**A**) Schematic of the *SUS1* gene structure. Asterisks denote non-canonical splice site sequences. Bold texts represent non-canonical bases, and the branch point (BP) adenosine is underlined. (**B**–**D**) RNA isolated from wild-type and *swt21*Δ strains was reverse-transcribed using a random primer. The synthesized cDNA was amplified by PCR with region-specific primers designed to analyze alternative splicing events in *SUS1*, including intron retention (**B**,**C**) and exon skipping (**D**). The percentage of intron retention is indicated below the corresponding lane for each strain. Primer locations are indicated by arrows in the panels. *SCR1* was used as a loading control to verify RNA integrity and equal input. Block diagrams above the gels in B–D represent alternative splicing isoforms, with color and order matching exon organization in the *SUS1* schematic.

As illustrated in Figure 1B,C, *SUS1* (with non-canonical 5′ss and BPS) was most severely affected, with a marked accumulation of the partly spliced intermediate. This was followed by *AML1* (with non-canonical 5′ss), whereas *DYN2* (with non-canonical 3′ss) showed only minimal accumulation (Figure S1D). Given the severe splicing defect in *SUS1* in the *swt21*Δ strain, splicing details were examined using RT-PCR with specific primers. *SUS1*, a component of the histone H2B ubiquitin protease system, is required for the establishment of proper chromatin modification states [23,24]. The *SUS1* gene exhibits a unique structure, containing two introns, with the first intron featuring a non-consensus 5′ss (GUAUGA) and a non-consensus BPS (UACUGAC) (Figure 2A) [25].

*SWT21* deletion promoted the accumulation of intron 1 in the *swt21*Δ strain (37.40%) compared to wild-type (14.67%), accompanied by a marked decrease in spliced mRNA levels (Figure 2B). Conversely, intron 2 retention was minimal, with no significant difference observed between the two strains (2.21% in *swt21*Δ vs. 1.62% in wild-type) (Figure 2C). Additionally, minimal exon 2 skipping was detected in the *swt21*Δ strain under prolonged exposure (Figure 2D), although the change was too subtle to be accurately quantified. These findings collectively suggest that Swt21p may contribute to the efficient recognition of non-consensus splice sites in *SUS1*, supporting its proper splicing. These results are consistent with those of prior studies, which have concluded that the deletion of core spliceosome components results in changes to splicing patterns, including intron retention and exon skipping in *SUS1* [26]. As previously reported, a short heat shock leads to increased retention of intron 1 in *SUS1* [26]. To assess Swt21p’s role under these conditions, we analyzed *SUS1* splicing after shifting cells to 42 °C for 30 min. No significant increase in intron 1 retention attributable to Swt21p deletion was observed under heat stress conditions (Figure S2).

### 2.3. SWT21 Deletion Reduces U1 snRNP Levels in the Pre-B Complex

**Figure 3:**
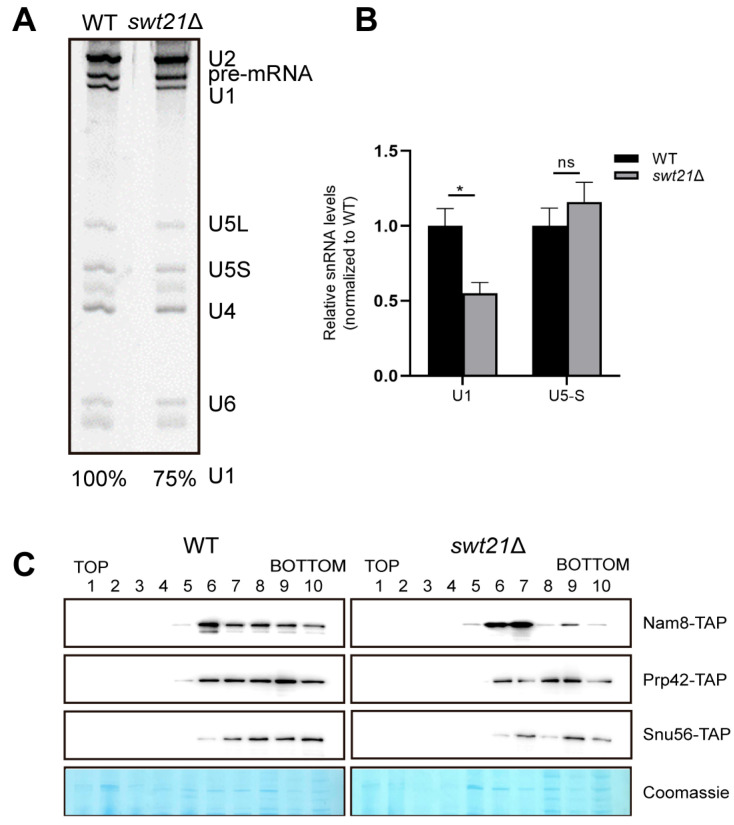
Deletion of *SWT21* reduces U1 snRNP levels in the pre-B complex. Spliceosomal complexes were assembled under in vitro splicing conditions using MS2-Actin pre-mRNA, MS2-MBP protein, and extracts from wild-type or *swt21*Δ *S. cerevisiae* cells. Affinity-purified spliceosomal complexes were subjected to glycerol gradient sedimentation. (**A**) The RNA composition of wild-type and *swt21*Δ spliceosomal complexes was analyzed by denaturing polyacrylamide gel electrophoresis (PAGE), with pre-mRNA and associated snRNAs (U1, U2, U4, U5, and U6) indicated in the legend. The level of U1 snRNA in each lane was quantified relative to the pre-mRNA band, which served as a spliceosome loading control, and normalized to the level of U1 snRNA in the WT (set to 100%). (**B**) RT-qPCR analysis of U1 and U5-S snRNAs in the 9th gradient fraction. Data are expressed as mean ± S.E.M. from at least three technical replicates. Statistical significance was determined using Student’s unpaired *t*-test (* *p* < 0.05; ns, not significant). (**C**) Proteins from all gradient fractions were analyzed by Western blotting to detect TAP-tagged proteins (Prp42-TAP, Nam8-TAP, Snu56-TAP). Coomassie staining was used as a loading control to verify equal protein input.

Considering the functional links between Swt21p and U1 snRNP, we then investigated whether the aforementioned splicing effects could result from Swt21p’s involvement in spliceosome assembly. The pre-B complex represents a key intermediate in spliceosome assembly, during which 5′ss is still recognized by the U1 snRNP [4]. Spliceosomes were assembled on Actin pre-mRNA under low ATP conditions, arresting the spliceosome at the pre-B complex stage [27,28]. Spliceosomes were affinity-purified and subsequently fractionated by glycerol gradient centrifugation. Then, the fractions were analyzed by denaturing polyacrylamide gel electrophoresis (PAGE), identifying fractions 8–10 as pre-B complexes based on their composition, which included U1, U2, U4, U5, and U6 snRNAs, as well as unspliced Actin pre-mRNA (Figure S3). Further analysis of fraction 9, where key spliceosomal components were enriched, unveiled that pre-B complexes formed in *swt21*Δ extracts retained substantial levels of U1, albeit at approximately 75% of the levels observed in wild-type (Figure 3A). RT-qPCR analysis validated this finding, showing a moderate reduction in U1 snRNA levels in *swt21*Δ pre-B complexes, while U5-S levels remained unchanged (Figure 3B).

The protein composition of spliceosomes assembled in the *swt21*Δ extracts was analyzed by mass spectrometry (MS). As expected, U1 snRNP proteins, including Nam8p, Snu56p, and Prp42p, exhibited reduced enrichment in pre-B complexes, while core proteins such as SmBp displayed only mild changes (Table S3). This may be attributed to the presence of these core proteins in other snRNPs, such as U2 and U4, which potentially masked the effects of *SWT21* deletion. Consistently, Western blot analysis uncovered decreased levels of Prp42p, Snu56p, and Nam8p in *swt21*Δ pre-B complexes compared to wild-type counterparts (fractions 8–10) (Figure 3C), with total protein loading as an internal reference. In contrast, the levels of other proteins typically associated with the pre-B complexes, such as U2 snRNP and U4/U6.U5 tri-snRNP, were comparable in both wild-type and *swt21*Δ complexes, as determined by peptide count analysis (Table S3).

**Figure 4:**
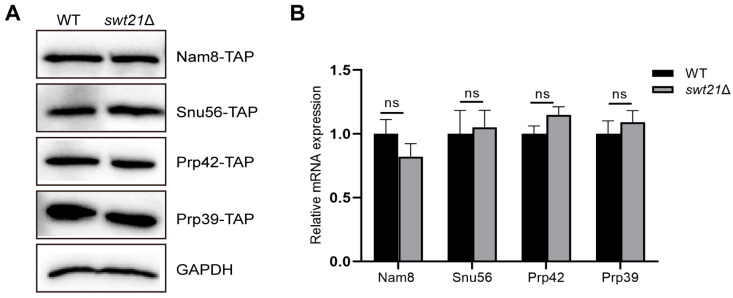
*SWT21* deletion does not affect the expression levels of U1 snRNP components. (**A**) Western blot analysis was performed on total protein from wild-type and *swt21*Δ strains expressing TAP-tagged Prp42, Nam8, Snu56, and Prp39. GAPDH served as a loading control. (**B**) Relative mRNA expression levels of Nam8, Snu56, Prp42, and Prp39 in wild-type and *swt21*Δ strains were determined using RT-qPCR. Data are presented as mean ± S.E.M. from at least three technical replicates. Statistical significance was determined by Student’s unpaired *t*-test (ns, not significant).

The observed reduction in the levels of U1 snRNP proteins within the pre-B complexes following *SWT21* deletion may be attributed to the instability of U1 snRNP during this stage, leading to dissociation or a decrease in the total cellular levels of U1-associated proteins. To distinguish between these possibilities, the cellular expression of U1 proteins and their corresponding mRNAs was investigated in wild-type and *swt21*Δ strains. Western blot analysis revealed comparable protein levels of U1 snRNP components between the strains (Figure 4A). Likewise, RT-qPCR revealed no changes in the corresponding mRNA levels (Figure 4B). These results conjointly indicate that the reduced levels of U1 snRNP proteins within the pre-B complexes in *swt21*Δ extracts were more likely attributable to U1 snRNP instability rather than a decrease in protein expression levels.

### 2.4. SWT21 Deletion Alters the Stability and Localization of the U1 Component Nam8p

**Figure 5:**
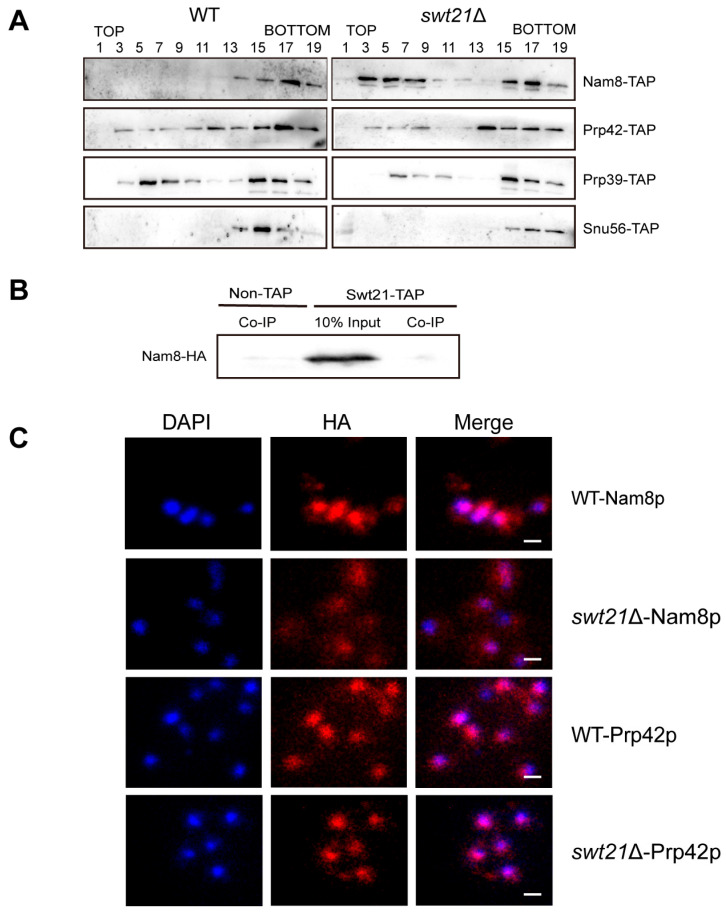
*SWT21* deletion causes partial dissociation of Nam8p from U1 snRNP and alters its cellular localization. (**A**) Glycerol gradient fractionation was used to analyze the distribution of Prp42-TAP, Prp39-TAP, Snu56-TAP, and Nam8-TAP in wild-type and *swt21*Δ strains. Cell extracts (without ATP or pre-mRNA) were subjected to a 10–30% gradient, and alternate fractions were analyzed by Western blotting using anti-PAP antibodies. (**B**) Extracts from Non-TAP and Swt21-TAP strains carrying Nam8-HA (without ATP or pre-mRNA) were incubated with IgG-agarose, and precipitates were analyzed by Western blotting using anti-HA antibodies. IP: immunoprecipitation. (**C**) Immunofluorescent localization of Nam8-HA and Prp42-HA in wild-type and *swt21*Δ cells. Nam8-HA and Prp42-HA were visualized as red fluorescent signals (anti-HA staining), with nuclei stained in blue (DAPI). Merged images are shown. The scale bars represent 10 μm.

To further assess the impact of Swt21p on U1 snRNP stability, the distribution of its components was analyzed in wild-type and *swt21*Δ cell extracts using glycerol gradient fractionation, followed by Western blot analysis. In wild-type extracts, Nam8p was primarily detected in higher-density fractions (Figure 5A, lanes 13–19), indicating its stable association with U1 snRNP [29]. In contrast, Nam8p exhibited a broader distribution in *swt21*Δ extracts, extending into lower-density fractions (lanes 3–7). This shift implied that, in the absence of Swt21p, a portion of Nam8p dissociated from U1 snRNP and transitioned into a free state. Interestingly, the distribution profiles of other spliceosomal proteins, such as Prp42p, Prp39p, and Snu56p, were not significantly altered in *swt21*Δ extracts, though minor dissociation could not be ruled out. These findings indicate that Swt21p is required for preserving Nam8p stability within the U1 snRNP.

To elucidate the physical association between Swt21p and Nam8p, co-immunoprecipitation (Co-IP) experiments were performed. No direct interaction was detected (Figure 5B), suggesting that Swt21p may indirectly stabilize the interaction between Nam8p and U1 snRNP. Furthermore, the localization of Nam8p was examined in *swt21*Δ cells. HA-tagged Nam8p was visualized using indirect immunofluorescence. In wild-type cells, Nam8p was localized in both the nucleus and cytoplasm. However, in *swt21*Δ cells, its localization partially shifted to the cytoplasm (Figure 5C). In contrast, the localization of Prp42p remained unchanged. This finding is consistent with the release of Nam8p from the complex in the absence of Swt21p.

### 2.5. Swt21p Acts as a Regulatory Factor in Spliceosome Assembly

To further explore the mechanism by which Swt21p contributes to spliceosome assembly, its association with early spliceosomal complexes was explored. The results revealed no detectable Swt21p in the pre-B complex (Figure S4; Table S3), indicating that any potential association with early spliceosomal complexes is either weak or highly transient.

**Figure 6:**
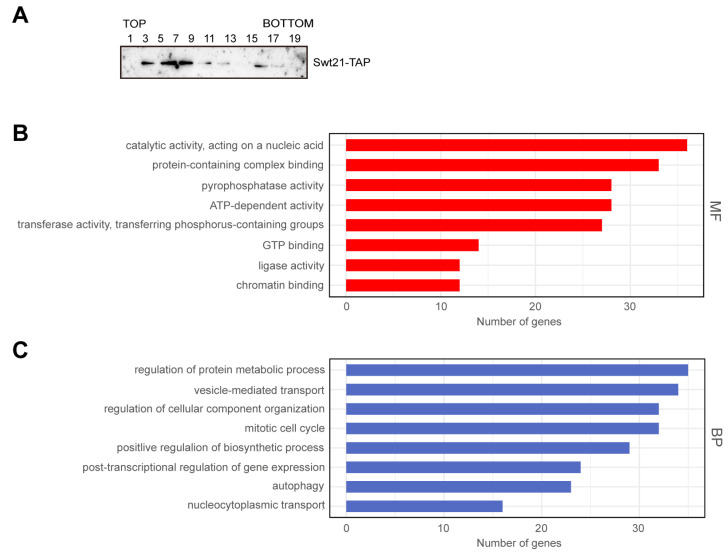
Swt21p predominantly exists in a free state and is involved in other cellular processes. (**A**) Gradient fractions from wild-type extracts expressing Swt21-TAP. Alternate gradient fractions were collected and analyzed by Western blotting using anti-PAP antibodies. (**B**,**C**) Swt21p-associated proteins were purified using TAP and analyzed by mass spectrometry. Gene Ontology (GO) analysis of the identified proteins was performed using ClusterProfiler (version 4.0), focusing on molecular function (**B**) and biological process (**C**). Detailed GO terms are presented in Table S5. The TAP-tagged Swt21p was functionally validated to ensure data reliability (Figure S6).

Next, the distribution of Swt21p was investigated by glycerol gradient fractionation, exposing that it was predominantly localized in lower-density fractions (Figure 6A, lanes 3–7), with minimal presence in higher-density fractions. This distribution pattern indicates that Swt21p primarily exists in an unbound state rather than being strongly associated with larger complexes. Meanwhile, Swt21p was detected only after prolonged exposure, consistent with its low cellular abundance, as previously reported [30].

Subsequently, tandem affinity purification (TAP) [31] and mass spectrometry were employed to identify Swt21p-associated proteins. In line with the findings of a previous study [32], the results corroborated that Swt21p does not directly interact with essential splicing factors, such as U snRNA splicing factors, supporting its classification as a non-core component of the spliceosome (Figure S5; Table S4). Taken together, these findings suggest that Swt21p serves as a regulatory factor in spliceosome assembly.

Of note, proteins co-purified with Swt21p were involved in various cellular processes (Figure S5; Table S4). Gene Ontology analysis of Swt21p-associated proteins revealed molecular functions, including pyrophosphatase activity, ATP-dependent activity, and chromatin binding (Figure 6B). In terms of biological processes, these proteins were linked to the mitotic cell cycle and post-transcriptional regulation of gene expression (Figure 6C). Collectively, these findings indicate that the function of Swt21p is not restricted to splicing and may extend to other cellular processes.

## 3. Discussion

Although the stable interaction of U1 snRNP with pre-mRNA during spliceosome assembly is principally driven by U1 components, auxiliary factors also play vital roles in this regulatory process [9,15,18]. However, the mechanisms by which these factors coordinate with U1 snRNP to facilitate spliceosome assembly remain underexplored. Herein, spliceosome affinity purification, glycerol gradient analysis, and TAP were employed to elucidate the role of Swt21p in regulating U1 snRNP during the early stages of spliceosome assembly in *S. cerevisiae*.

In this study, Swt21p was not essential for yeast viability but impaired the efficiency of pre-mRNA splicing, with distinct effects in vivo and in vitro. At the same time, RT-PCR analysis demonstrated that while *swt21*Δ did not affect the splicing of single-intron genes in vivo, it specifically impaired the splicing of multi-intron genes, particularly *SUS1*. This functional is likely due to the structural complexity of *SUS1*, which contains two distinct introns. The first intron has a non-canonical 5′ss (GUAUGA) and BPS (UACUGAC), whereas the second intron contains canonical splice sites [33]. Further investigation showed that *SWT21* deletion altered the splicing pattern of *SUS1*, resulting in higher retention of the first intron and a marginal level of exon skipping (Figure 2B–D). Similar results were observed upon deletion of core spliceosome components, such as Nam8p and Lea1p [26]. More importantly, these observations imply that Swt21p may play a similar auxiliary role in facilitating the recognition of non-consensus splice sites in *SUS1*. In contrast to the two-intron *SUS1* RNA, we also examined the impact of *SWT21* deletion on alternatively spliced transcripts that contain only a single intron, including *MER2*, *PTC7*, *RPL30*, and *YRA1* [34,35,36,37]. Notably, only *PTC7*, which produces isoforms via intron retention [37], exhibited impaired splicing in the *swt21*Δ strain (Figure S7). Furthermore, in vitro assays revealed that Swt21p acts as a general splicing factor, with its absence leading to more severe splicing defects. One possibility is that compensatory splicing factors or regulatory mechanisms present in vivo alleviate the splicing defects induced by *SWT21* deletion. In contrast, such mechanisms are absent in vitro, where the simplified environment may expose a broader requirement for Swt21p [38].

Our findings reveal a previously uncharacterized role for Swt21p in the early stages of spliceosome assembly. Specifically, a reduction in U1 snRNP levels was noted within the pre-B complex formed in the absence of Swt21p (Figure 3A–C), suggesting the compromised stability of U1 snRNP during this stage. These results are in agreement with previous genetic observations that *SWT21* deletion circumvents the requirement for Prp28p [18], a DEAD-box ATPase that acts on the pre-B complex to displace U1 snRNP from the 5′ss [39]. It is worthwhile emphasizing that structural studies further support our findings by establishing that the pre-B complex exhibits a flexible and asymmetric architecture, where U1 and U2 snRNPs engage in weak and transient interactions with each other and with the tri-snRNP [40]. This fragile interface may account for the enhanced destabilization of U1 snRNP observed upon *SWT21* deletion. Altogether, these findings highlight the role of Swt21p in stabilizing the interaction between U1 snRNP and the spliceosome. Correspondingly, Swt21p has been shown to promote the formation of the stable commitment complex (CC) [18]. However, the effect of *SWT21* deletion on U1 snRNP is stage-specific. In the absence of Swt21p, only the levels of a few core proteins, such as SmBp, are reduced in the CC, whereas those of most U1 snRNP components are decreased in the pre-B complex. This discrepancy likely arises from the transition between CC and pre-B, during which extensive structural rearrangements occur, including the incorporation of U2 snRNP, tri-snRNP, and other associated factors [40,41]. These findings support the hypothesis that Swt21p promotes early spliceosome assembly by maintaining U1 snRNP stability during the formation of the pre-B complex.

Considering that Swt21p specifically affects U1 snRNP within the pre-B complex, we postulate that it may influence the stability of U1 snRNP itself. Glycerol gradient analysis of U1 snRNP components and TAP purification assays validate this hypothesis. Deletion of *SWT21* causes partial dissociation of Nam8p from U1 snRNP, leading to increased free Nam8p levels in vivo (Figure 5A). Nam8p, an intrinsic RNA-binding component of the U1 snRNP, directly binds pre-mRNA at the 5′ss to stabilize U1/5′ss interactions [42] and maintain associations with U1 snRNP proteins such as Snu56p and Snu71p [11]. Nam8p dissociation in *swt21*Δ likely disrupts these interactions, compromising U1 snRNP assembly. Consistently, we found partial cytoplasmic accumulation of Nam8p in *swt21*Δ cells, but the underlying mechanism remains unclear and requires further investigation (Figure 5C). Additionally, Nam8-TAP purification assays showed that several U1 snRNP proteins, including Luc7p, Snu71p, and SmBp, were enriched in the absence of Swt21p (Figure S8, Table S6), indicating a compensatory accumulation of these proteins for maintaining U1 snRNP stability. This might explain why *SWT21* deletion does not alter U1 snRNP mobility on native gels [18] and impairs the splicing of multi-intron genes in vivo. These findings indicate that Swt21p regulates the interactions between U1 snRNP components, possibly through Nam8p, thereby ensuring the stability of U1 snRNP during early spliceosome assembly. However, no direct interaction between Swt21p and Nam8p was detected in Co-IP assays (Figure 5B), although the possibility of transient or weak interactions cannot be excluded.

This study defines Swt21p as a spliceosome assembly regulator rather than a core component, which is supported by several findings: (i) Glycerol gradient centrifugation indicated that Swt21p is primarily in a free state (Figure 6A); (ii) Swt21p was not detected in the pre-B complex (Figure S4; Table S3), confirming that it is not a stable component of the pre-B complex; (iii) TAP combined with MS demonstrated that Swt21p does not associate with essential splicing factors, including U snRNA-binding proteins (Figure S5; Table S4). These findings are consistent with those of previous studies concluding that Swt21p does not cross-link to pre-mRNA and is not an integral component of the core commitment complex [43]. Overall, these results reinforce the regulatory role of Swt21p in spliceosome assembly. Swt21-TAP purification analysis suggests that Swt21p may also participate in other functions such as phosphorylation. Phosphorylation of splicing factors, such as TIA1 (the human Nam8 homolog), promotes U1 snRNP recruitment to the 5′ splice site [44]. A plausible scenario is that Swt21p serves as an adapter for recruiting protein kinases to modify spliceosomal components, such as Nam8p, thus ensuring their proper function.

**Figure 7:**
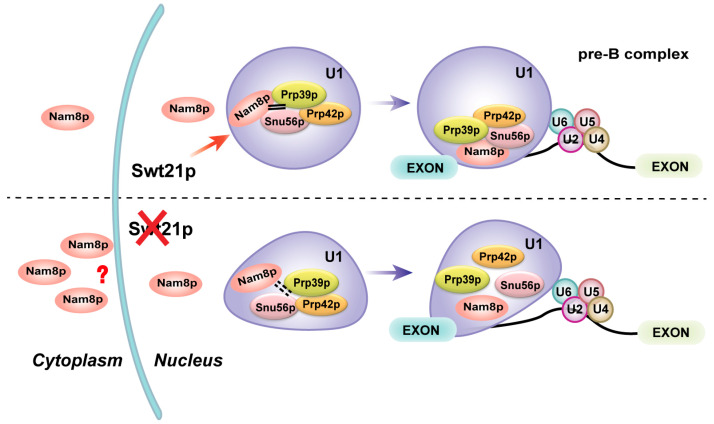
Model: A role for Swt21p in the early stage of spliceosome assembly. Under wild-type conditions, Swt21p regulates the interactions between U1 snRNP components, potentially through Nam8p, thereby ensuring the stability of U1 snRNP within the pre-B complex. In the absence of Swt21p (indicated by the red cross), Nam8p partially dissociates from the U1 snRNP into a free state, accompanied by its altered localization (how this cytoplasmic accumulation occurs remains unclear; indicated by the red question mark). This disruption may impair U1 snRNP assembly or destabilize its association, thereby preventing proper pre-B complex formation or causing its premature disassembly. Consequently, this disruption leads to splicing defects. The red arrow indicates the regulatory role of Swt21p, and the purple arrow depicts a simplified transition from U1 snRNP to the pre-B complex during early spliceosome assembly.

In summary, we propose a model in which Swt21p plays a regulatory role in coordinating the interactions between U1 snRNP components, possibly through Nam8p, thereby ensuring the stability of U1 snRNP within the pre-B complex. In the absence of Swt21p, Nam8p partially dissociates from the U1 snRNP and mislocalizes, potentially disrupting U1 snRNP assembly or destabilizing its association, which in turn affects the proper formation of the pre-B complex or contributes to its premature disassembly (Figure 7). Consequently, these disruptions may result in splicing defects. Our study highlights the important function of Swt21p in early spliceosome assembly while also suggesting that Swt21p may have additional functions beyond splicing.

## 4. Materials and Methods

### 4.1. Yeast Strains, Plasmids, and Primers

The *S*. *cerevisiae* strains, plasmids, and primer sequences used in this study are summarized in Tables S1 and S2. Strains harboring deletions or TAP/HA tags were constructed as described in earlier studies [45,46]. Yeast strains were grown in YP medium supplemented with 2% glucose (YPD), or in selective synthetic dropout media (SD–URA or SD–HIS), at 25 °C unless otherwise indicated.

### 4.2. Splicing Extracts and In Vivo and In Vitro Splicing

Splicing extracts were prepared by grinding frozen cells in liquid nitrogen [47]. Yeast cells were harvested at the indicated growth phase (OD_600_ −4.0 for splicing reaction; −2.0 for TAP purification) and lysed in liquid nitrogen by cryogenic grinding in AGK buffer (20 mM HEPES/KOH pH 7.9, 200 mM KCl, 1.5 mM MgCl_2_, 8% glycerol) supplemented with protease inhibitors. The lysate was clarified by centrifugation at 17,000 rpm for 30 min at 4 °C. The resulting supernatant was then subjected to ultracentrifugation at 48,000 rpm for 1 h at 4 °C in a Hitachi S58A rotor, and the middle phase was collected. Extracts were dialyzed twice at 4 °C against Buffer D (20 mM HEPES/KOH pH 7.9, 50 mM KCl, 0.2 mM EDTA, 0.4 mM MgCl_2_, 20% glycerol, 0.5 mM DTT, 0.5 mM PMSF, 2 mM benzamidine) for 1.5 h each. The second dialysis was performed in Buffer D lacking benzamidine. Final extracts were aliquoted and stored at −80 °C.

To assess in vivo splicing efficiency, strains were cultured to an OD_600_ of 2.0 and harvested for total RNA extraction using the hot phenol method, followed by DNase I treatment. cDNA synthesis was performed using the ProtoScript First Strand Kit (New England Biolabs, Ipswich, MA, USA) and random primers, then amplified via PCR with specific primers. Next, PCR products were separated on a native agarose gel, visualized with Gel Red (US Everbright Inc., Suzhou, China), and quantified using ImageJ (version 1.54p) for analysis.

In vitro, the splicing reaction was assembled as follows. MS2-pre-mRNA, containing three tandem phage R17 MS2-binding sites, was transcribed in vitro using T7 RNA polymerase. The reaction mixture consisted of 20% dialyzed wild-type or *swt21*Δ extracts, 1.8 nM MS2-pre-mRNA, 54 nM MS2-MBP protein, 60 mM K-phosphate buffer (pH 7.25), 0.3% (*w*/*v*) PEG8000, 2.5 mM MgCl_2_, 2 mM spermidine, and 2 mM ATP. After incubation at 23 °C for 1 h, the mixture was loaded onto an amylose–agarose column (NEB). RNA was extracted from the eluate and analyzed as described above.

### 4.3. Affinity Purification of Yeast Pre-B Complex

To prepare the pre-B complex, MS2-Actin pre-mRNA was incubated with MS2-MBP fusion protein for 1 h at 4 °C. The complex was subsequently mixed with splicing extracts under standard in vitro conditions to stall splicing at the pre-B complex stage [28]. Afterward, the reaction mixture was applied to an amylose–agarose column pre-equilibrated with GK75 buffer (20 mM HEPES pH 7.25, 75 mM KCl, 1.5 mM MgCl_2_, 0.2 mM EDTA pH 8.0). After washing, spliceosomes were eluted with 100 mM maltose in GK75 and loaded onto a 10–30% (*v*/*v*) glycerol gradient. The gradient was centrifuged at 55,000 rpm for 2.5 h in a SW60Ti rotor, and 10 fractions of 400 μL each were collected. RNAs were extracted using phenol/chloroform extraction followed by ethanol precipitation and analyzed by 8% denaturing PAGE or RT-qPCR. Proteins were extracted using TCA precipitation and analyzed by Western blotting and mass spectrometry (MS).

### 4.4. Quantitative Real-Time Polymerase Chain Reaction (qPCR)

Quantitative real-time PCR was carried out on the QuantStudio^TM^ 6 Flex detection system (Thermo Fisher Scientific, Waltham, MA, USA) with the TB Green Premix Ex Taq II (Tli RNaseH Plus; TaKaRa, Tokyo, Japan). Relative expression levels of target genes were quantified using the 2^−ΔΔCt^ method, with actin serving as the internal reference for normalization. All experiments were performed independently in duplicates or triplicates.

### 4.5. Protein Sample Preparation and Western Blot Analysis

Protein samples were extracted using a NaOH lysis protocol followed by trichloroacetic acid (TCA) precipitation [48]. For Western blot analysis, proteins were separated on a 4–12% Bis-Tris gel (GenScript) using Tris-MOPS buffer, transferred onto a polyvinylidene fluoride (PVDF) membrane, and incubated with anti-PAP antibodies ( P1291, Sigma-Aldrich, St. Louis, MO, USA). Antibody-bound signals were detected using ECL reagents (Tanon, Shanghai, China).

### 4.6. Glycerol Gradient Analysis for the Distribution of Spliceosome Components

Glycerol gradient analysis was performed as outlined in a previous study [49]. TAP-tagged protein extracts (80 µL) were mixed with 120 µL of GC buffer (20 mM HEPES pH 7.0, 100 mM KCl, 0.2 mM EDTA) to achieve a final glycerol concentration of 8%. Thereafter, the sample was loaded onto a 10–30% glycerol gradient and centrifuged at 55,000 rpm for 6 h at 4 °C in a SW60 Ti rotor (Beckman, Indianapolis, IN, USA). The gradient was fractionated into 20 equal volumes of 200 µL. Alternate fractions were subjected to trichloroacetic acid (TCA) precipitation and analyzed by Western blotting.

### 4.7. Immunoprecipitation Assay and Immunofluorescence Microscopy

Immunoprecipitation assays were performed [50] using IPP300 buffer and IgG Sepharose 6 Fast Flow (GE Healthcare, Chicago, IL, USA). Extracts from Non-TAP and Swt21-TAP strains carrying Nam8-HA (50 μL) were incubated with the beads on a rotating mixer for 2 h at 4 °C. After washing with IPP300 buffer, the pellet was resuspended in 20 μL 1× SDS loading buffer, vortexed, and heated at 95 °C for 5 min prior to Western blot analysis.

For immunofluorescence staining, HA-tagged Nam8p/Prp42p strains were cultured to an OD600 of 0.3–0.5 and fixed with 4% formaldehyde for 40 min at 25 °C. Cells were washed with Buffer B (0.1 M potassium phosphate pH 7.0, 1.2 M sorbitol) and resuspended in Buffer B containing Snail enzyme, followed by incubation for 2 h at 37 °C. After centrifugation, cells were placed on poly-L-lysine-coated slides, blocked with 5% milk in PBST, and incubated overnight with anti-HA antibody (1:1000, HUABIO) at 4 °C. After washing, cells were incubated with Alexa Fluor 594 secondary antibody (Proteintech Group) for 1 h at 23 °C in the dark, followed by DAPI staining. Images were captured using a fluorescence microscope (Nikon ECLIPSE Ti2, Nikon, Tokyo, Japan).

### 4.8. TAP Purification of Swt21p/Nam8p Complexes

Wild-type strains or strains expressing TAP-tagged Swt21p/Nam8p were cultured to an OD_600_ of 2.0. Whole-cell yeast extracts were collected and incubated with 200 μL IgG Sepharose 6 Fast Flow (GE Healthcare) for 2 h at 4 °C. After gravity elution of the supernatant, the beads were washed with 40 mL IPP300 Buffer (10 mM Tris-HCl pH 7.9, 300 mM NaCl, 0.1% NP40) and 20 mL CBB (calmodulin binding buffer; 25 mM Tris-HCl pH 7.9, 300 mM NaCl, 1 mM MgOAc_2_, 1 mM imidazole, 2 mM CaCl_2_, 2 mM DTT). The complex was eluted by incubation with 24 μL PreScission (1.2 mg/mL) in 2 mL CBB and 1 μL rRNasin overnight. The second purification step was performed by incubating the sample with 200 μL of calmodulin bead suspension (Calmodulin Affinity Resin, Agilent, Santa Clara, CA, USA) for 1 h at 4 °C. After washing with 40 mL CBB, the complex was eluted using 1 mL CEB (calmodulin elution buffer; 25 mM Tris pH 7.9, 300 mM NaCl, 1 mM MgOAc_2_, 1 mM imidazole, 25 mM EGTA, 0.02% NP-40, 2 mM DTT) for 5 min. One-fourth of the eluates were purified, resolved on a 4–12% Bis-Tris gel (GenScript, Nanjing, China) using Tris-MOPS buffer and silver-stained. Half of the eluates were used for mass spectrometry analysis. The samples were heat-denatured, reduced with TCEP, alkylated with IAA, and purified using the SP3 protocol with ethanol precipitation. After digestion with trypsin in 50 mM ammonium bicarbonate at 37 °C for 16 h, the resulting peptides were lyophilized and identified by mass spectrometry.

### 4.9. Mass Spectrometry and Gene Ontology Analysis

Proteins were identified by MS as described in a previous study [27]. Gene Ontology (GO) analysis was carried out using ClusterProfiler (version 4.0), and classification histograms were generated to visualize the distribution of enriched terms in biological processes (BP) and molecular functions (MF). GO pathways with *p* < 0.05 were considered significantly enriched.

## Data Availability

The original contributions presented in this study are included in the article/Supplementary Materials. Further inquiries can be directed to the corresponding author.

## Supplementary Materials

The following supporting information can be downloaded at: https://www.mdpi.com/article/10.3390/ijms26125440/s1.
