# Supplementary material for: Swt21p Is Required for Nam8p-U1 snRNP Association and Efficient Pre-mRNA Splicing in Saccharomyces cerevisiae
Source: Int J Mol Sci. 2025 Jun 6;26(12):5440. doi: 10.3390/ijms26125440 (PMC12192654; doi:10.3390/ijms26125440)
Supplement: Supplementary file 1 [file ijms-26-05440-s001.zip › Revised_Supplementary Figure Legends.pdf]

Supplementary figure legends

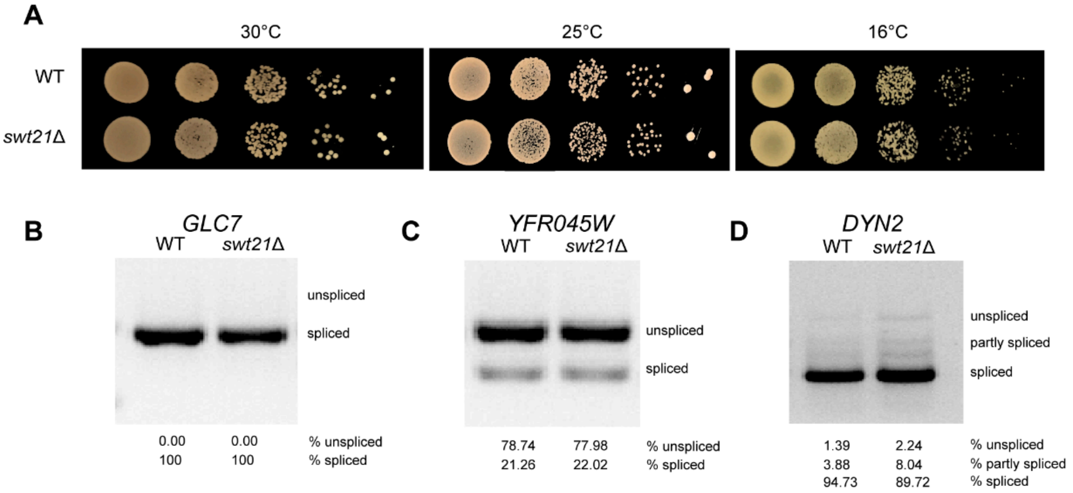

Figure S1. Swt21p does not affect yeast growth and promotes multi-intron splicing efficiency *in vivo*. (A) Growth analysis of wild-type and *swt21Δ* strains at different temperatures: Cultures were serially diluted, spotted on a solid medium, and incubated for 3 days (4 days at 16°C). (B-D) Swt21p facilitates efficient splicing of multi-intron genes *in vivo*. Total RNA from the wild-type and *swt21Δ* strains was reverse transcribed and PCR-amplified using gene-specific primers targeting the first and last exons of the *GLC7* (B), *YFR045W* (C), and *DYN2* (D) genes. PCR products were separated by agarose gel electrophoresis, visualized with Gel Red staining, and the RT-PCR products of unspliced, partly spliced, and fully spliced transcripts are indicated on the right. Percentages of each splicing product were quantified and are displayed below each corresponding lane.

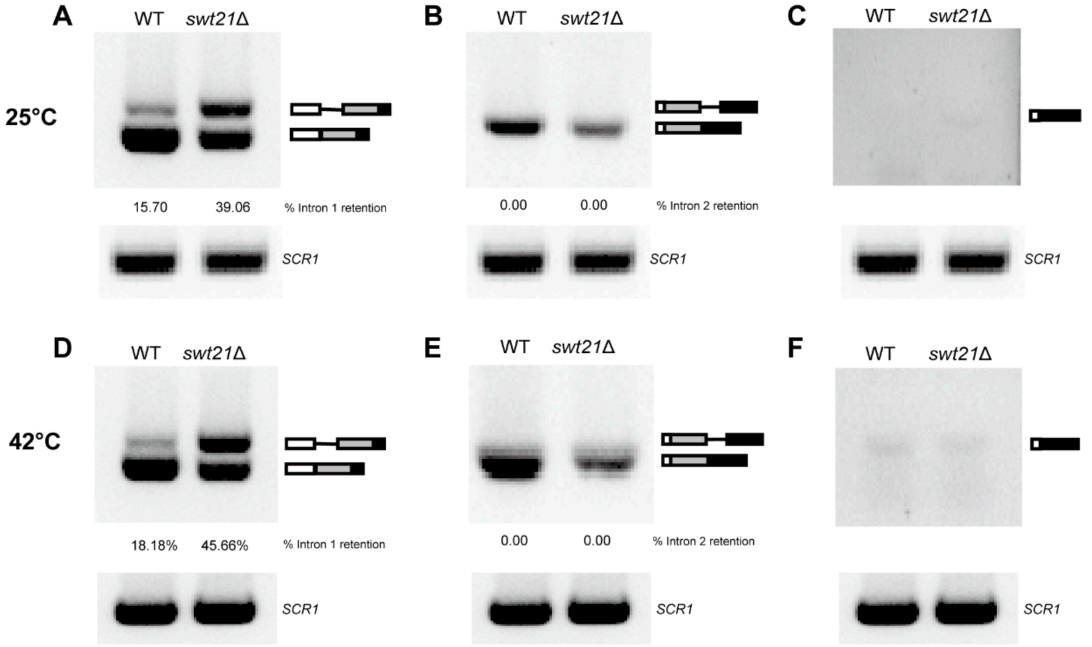

Figure S2. *SWT21* deletion does not significantly alter heat shock–induced intron 1 retention in *SUS1* pre-mRNA. (A–C) RT–PCR analysis of *SUS1* splicing in WT and *swt21Δ* cells grown at 25°C. (D–F) RT–PCR analysis following a 30-minute shift from 25°C to 42°C. Total RNA was isolated and analyzed for splicing isoforms associated with intron 1 retention (A, D), intron 2 retention (B, E), and exon 2 skipping (C, F). Stick diagrams on the right illustrate the corresponding isoforms. The percentage of intron

retention is indicated below the corresponding lane for each strain. *SCR1* was used as a loading control.

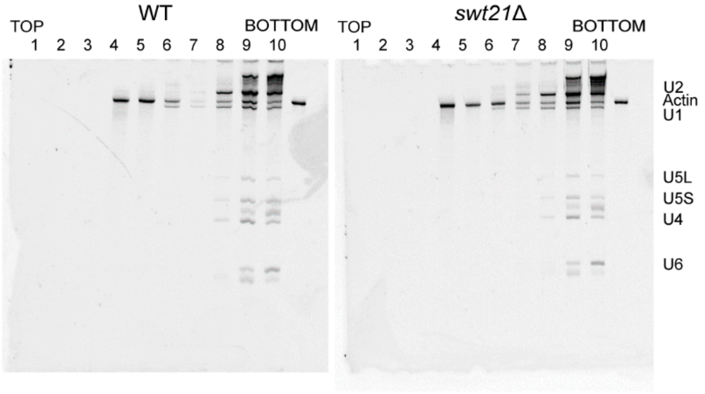

Figure S3. RNA composition of spliceosomal complexes in glycerol gradient fractions. Spliceosomal complexes were assembled *in vitro* using MS2-MBP protein, MS2-Actin pre-mRNA, and extracts from wild-type or *swt21Δ S. cerevisiae* cells. Affinity-purified complexes were subjected to glycerol gradient sedimentation, and RNA compositions of all fractions were analyzed by denaturing polyacrylamide gel electrophoresis.

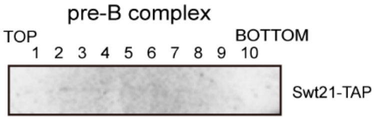

Figure S4. Swt21p is not detected in the pre-B complex. Spliceosomal complexes were assembled *in vitro* using MS2-MBP protein, MS2-Actin pre-mRNA, and extracts from wild-type *S. cerevisiae* cells. Affinity-purified complexes were subjected to glycerol gradient sedimentation. Proteins from all gradient fractions were analyzed by western blotting to detect Swt21-TAP.

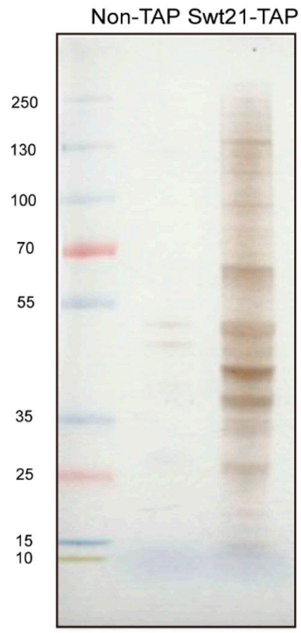

Figure S5. Proteins co-purifying with TAP-tagged Swt21p. TAP-purified proteins were separated on a 4%-12% Bis-Tris gel and visualized via silver staining. Protein identities were determined by mass spectrometry. Detailed protein identifications are provided in Supplementary Table S4.

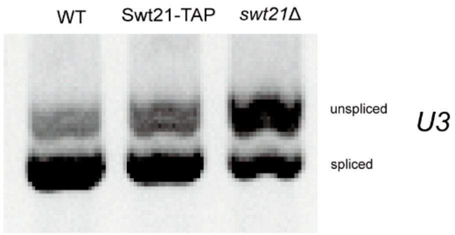

Figure S6. Functional analysis of TAP-tagged Swt21p using *in vitro U3* splicing assay. Splicing reactions were carried out at 23°C using cell extracts prepared from wild-type (WT), Swt21p-TAP, and *swt21Δ* strains. RNA was extracted by phenol–chloroform followed by ethanol precipitation, and splicing efficiency was analyzed by RT–PCR. The resulting unspliced andspliced products are indicated on the right.

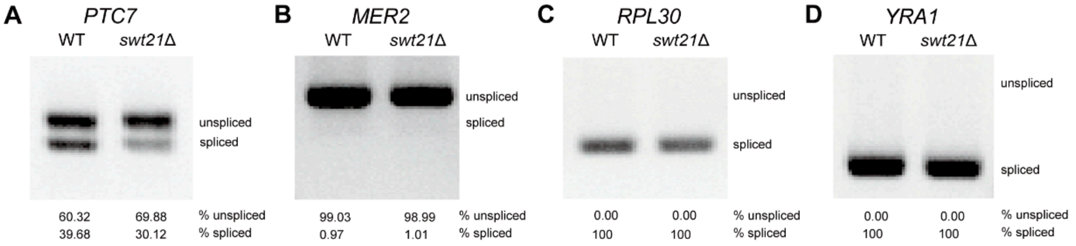

Figure S7. RT-PCR analysis of alternatively spliced transcripts with a single intron in *swt21Δ* cells.. Total RNA from wild-type (WT) and *swt21Δ* strains was reverse transcribed using random primers, and cDNA was amplified by PCR with gene-specific primers for the first and last exons of *PTC7* (A), *MER2* (B), *RPL30*

(C) and *YRA1* (D). PCR products were separated by native agarose gel electrophoresis and detected using Gel Red staining. The unspliced and spliced RT-PCR products are labeled on the right, with their quantified percentages shown below each lane.

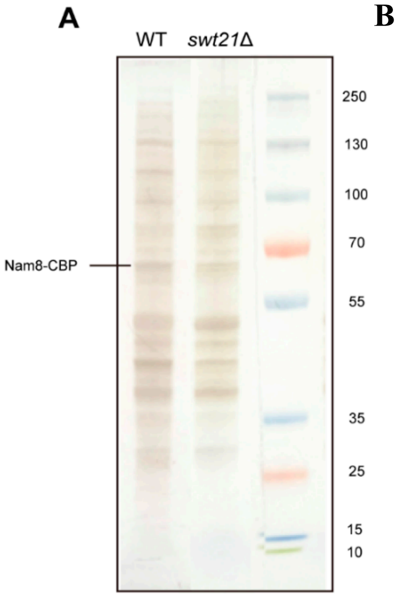

B

	Proteins	WT (Spectrum count)	*Swt21*Δ (Spectrum count)
U1 snRNP	**Nam8**	**38**	**19**
	Prp39	53	21
	**Snu71**	**30**	**73**
	**Prp40**	**106**	**161**
	Prp42	39	12
	Snu56	36	13
	Snp1 (RU17)	23	22
	Mud1 (RU1A)	15	20
	**Luc7**	**7**	**19**
	Yhc (RU1C)	15	8
	**B (RSMB)**	**6**	**40**
	**D1 (SMD1)**	**10**	**33**
	**D2**	**1**	**18**
	D3	3	1

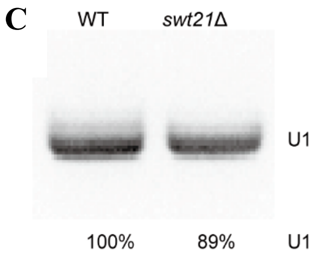

Figure S8. Proteins co-purifying with TAP-tagged Nam8p. Profiles of proteins purified from wild-type (WT) and *swt21*Δ strains expressing TAP-tagged Nam8p using TAP purification. (A) Proteins were separated on a 4%-12% Bis-Tris gel and detected by silver staining. (B) Protein identities were determined by mass spectrometry. Spectral counts of U1 snRNP components in Nam8-TAP complexes from WT and *swt21*Δ strains. The relative enrichment of specific proteins was determined by comparing the spectrum counts of each protein to that of Nam8p. Higher ratios of protein/Nam8p spectrum counts in *swt21*Δ indicate greater enrichment of those proteins in the absence of Swt21p. See Supplementary Table S6 for details. (C) Total RNA was extracted from Nam8-TAP–purified eluates, followed by reverse transcription. U1 snRNA levels were assessed by RT–PCR to evaluate and normalize potential differences in U1 snRNP recovery between wild-type (WT) and *swt21*Δ strains.

Table S1. List of yeast strains and plasmids used in this study
Table S2. List of primers used in this study
Table S3. Protein composition of *S.cerevisiae* wild-type and *swt21*Δ pre-B spliceosomal complexes
Table S4. Proteins co-purifying with TAP-tagged Swt21p
Table S5. GO analysis
Table S6. Proteins co-purifying with TAP-tagged Nam8p in wild-type and *swt21*Δ strains
