# Supplementary material for: Swt21p Is Required for Nam8p-U1 snRNP Association and Efficient Pre-mRNA Splicing in Saccharomyces cerevisiae
Source: Int J Mol Sci. 2025 Jun 6;26(12):5440. doi: 10.3390/ijms26125440 (PMC12192654; doi:10.3390/ijms26125440)
Supplement: Supplementary file 1 [file ijms-26-05440-s001.zip › Table S1 List of yeast strains and plasmids used in this study.pdf]

Table S1. List of yeast strains and plasmids used in this study

Plasmid or strain	Purpose or genotype	Source
Strain		
Prp2-1	*(3.2 AID) MATalpha, prp2-1, ade2, his3, lys2-801, ura3*	R-Jlin
Plasmids		
pBS1539-psc	PCR template for TAP tag with *URA3* marker	Kum-Loong Boon
	PCR template for gene knockout with *URA3* marker	
pFA6a-HisMX6-3HA	PCR template for HA tag with *HIS* marker	Longtine et al. (46)
	PCR template for gene knockout with *HIS* marker	
