# Supplementary material for: Swt21p Is Required for Nam8p-U1 snRNP Association and Efficient Pre-mRNA Splicing in Saccharomyces cerevisiae
Source: Int J Mol Sci. 2025 Jun 6;26(12):5440. doi: 10.3390/ijms26125440 (PMC12192654; doi:10.3390/ijms26125440)
Supplement: Supplementary file 1 [file ijms-26-05440-s001.zip › Table S3 Protein composition of S.cerevisiae wt and swt21Δ pre-B spliceosomal complexes.pdf]

Table S3. Protein composition of *S. cerevisiae* wt and *swt21*Δ pre-B spliceosomal complexes

Yeast name protein	MW (kDa)	WT	*swt21*Δ
Sm proteins			
B(RSMB)	22.4	105	96
D1(SMD1)	16.3	138	92
D2	12.8	62	51
D3	11.2	171	94
E(RUXE)	10.4	14	10
F or Smx3(RUXF)	9.6	18	16
G or X2(RUXG)	8.5	18	11
U1 snRNP			
Prp39	74.7	134	63
Snu71	71.4	175	105
Prp40	69	237	126
Prp42	65	91	43
Nam8	56.9	107	63
Snu56	56.5	124	52
Snp1(RU17)	34.4	136	82
Mud1(RU1A)	34.4	141	66
Luc7	30	81	42
Yhc(RU1C)	27	91	38
U2 snRNP			
Rse1	153.8	256	159
Hsh155(SF3B1)	110	289	223
Prp9	63	233	154
Cus1	50.2	239	163
Prp21	33	172	107
Prp11	29.9	109	71
Lea1(RU2A)	27.2	135	91
Hsh49	24.5	117	72
Msl1	12.8	45	29
Rds3	12.3	22	11
Ysf3	10	37	20
U5 snRNP			
Prp8	279.5	566	513
Brr2	246.2	420	386
Snu114(Sn114)	114	229	223
Prp6	104.2	161	141
Prp28	66.6	0	0
Lin1	40.4	0	0
Dib1	16.7	19	22
U4/U6 snRNP			
Prp31	56.3	96	87
Prp3	56	176	147
Prp4	52.4	112	86
Snu13	13.6	6	5

U4/U6.U5 snRNP proteins			
Snu66	66.4	192	163
Sad1	52.2	0	0
Spp381	34	70	69
Prp38	28	33	32
Snu23	23	81	63
Lsm proteins			
Lsm4	21.3	41	37
Lsm7	13	58	34
Lsm8	12.4	20	15
Lsm2	11.2	38	26
Lsm5	10.4	11	10
Lsm3	10	11	9
Lsm6	9.4	7	7
RES complex			
Bud13(CWC26)	30.5	165	102
Pml1	23.6	47	28
Snu17/Ist3	17	46	25
Early splicing			
Prp5	96.4	87	27
Urn1	54	69	65
Swt21	40	0	0
