# Supplementary material for: Swt21p Is Required for Nam8p-U1 snRNP Association and Efficient Pre-mRNA Splicing in Saccharomyces cerevisiae
Source: Int J Mol Sci. 2025 Jun 6;26(12):5440. doi: 10.3390/ijms26125440 (PMC12192654; doi:10.3390/ijms26125440)
Supplement: Supplementary file 1 [file ijms-26-05440-s001.zip › Table S6 Proteins co-purifying with TAP-tagged Nam8p in wild-type (wt) and swt21Δ strains.pdf]

Table S6. Proteins co-purifying with TAP-tagged Nam8p in wild-type and *swt21*Δ strains

Yeast name protein	MW (kDa)	WT	*swt21*Δ
Nam8	56.9	38	19
Sm proteins			
B(RSMB)	22.4	6	40
D1(SMD1)	16.3	10	33
D2	12.8	1	18
D3	11.2	3	1
E(RUXE)	10.4	2	2
F or Smx3(RUXF)	9.6	0	0
G or X2(RUXG)	8.5	0	0
U1 snRNP			
Prp39	74.7	53	21
Snu71	71.4	30	73
Prp40	69	106	161
Prp42	65	39	12
Snu56	56.5	36	13
Snp1(RU17)	34.4	23	22
Mud1(RU1A)	34.4	15	20
Luc7	30	7	19
Yhc(RU1C)	27	15	8
U2 snRNP			
Mud2	60.5	1	0
Rse1	153.8	2	0
Hsh155	110	0	0
Prp9	63	1	0
Cus1	50.2	1	0
Prp21	33	0	0
Prp11 or RNA11	29.9	0	0
Lea1	27.2	0	0
Hsh49	24.5	0	0
Msl1	12.8	0	0
Rds3	12.3	0	0
Ysf3	10	0	0
U5 snRNP			
Prp8	279.5	4	1
Brr2	246.2	2	0
Snu114	114	0	0
Prp6	104.2	0	0
Prp28	66.6	2	0
Lin1	40.4	0	0
Dib1	16.7	1	0
U4/U6 snRNP			
Prp31	56.3	0	0
Prp3	56	0	0
Prp4	52.4	0	0

Snu13	13.6	13	6
Snu66	66.4	1	4
Sad1	52.2	1	0
Spp381	34	0	0
Prp38	28	0	0
Snu23	23	0	0
